# PREX1 integrates G protein-coupled receptor and phosphoinositide 3-kinase signaling to promote glioblastoma invasion

**DOI:** 10.18632/oncotarget.14348

**Published:** 2016-12-29

**Authors:** Alexander Gont, Manijeh Daneshmand, John Woulfe, Sylvie J. Lavictoire, Ian A.J . Lorimer

**Affiliations:** ^1^ Cancer Therapeutics Program, Ottawa Hospital Research Institute, Ottawa, Canada; ^2^ Department of Biochemistry, Microbiology and Immunology, University of Ottawa, Ottawa, Ontario, Canada; ^3^ Department of Pathology and Laboratory Medicine, University of Ottawa, Ottawa, Ontario, Canada; ^4^ Department of Medicine, University of Ottawa, Ottawa, Ontario, Canada

**Keywords:** glioma, glioblastoma, invasion, PREX1, dopamine receptor

## Abstract

A defining feature of the brain cancer glioblastoma is its highly invasive nature. When glioblastoma cells are isolated from patients using serum free conditions, they accurately recapitulate this invasive behaviour in animal models. The Rac subclass of Rho GTPases has been shown to promote invasive behaviour in glioblastoma cells isolated in this manner. However the guanine nucleotide exchange factors responsible for activating Rac in this context have not been characterized previously. PREX1 is a Rac guanine nucleotide exchange factor that is synergistically activated by binding of G protein αγ subunits and the phosphoinositide 3-kinase pathway second messenger phosphatidylinositol 3,4,5 trisphosphate. This makes it of particular interest in glioblastoma, as the phosphoinositide 3-kinase pathway is aberrantly activated by mutation in almost all cases. We show that PREX1 is expressed in glioblastoma cells isolated under serum-free conditions and in patient biopsies. PREX1 promotes the motility and invasion of glioblastoma cells, promoting Rac-mediated activation of p21-associated kinases and atypical PKC, which have established roles in cell motility. Glioblastoma cell motility was inhibited by either inhibition of phosphoinositide 3-kinase or inhibition of G protein βγ subunits. Motility was also inhibited by the generic dopamine receptor inhibitor haloperidol or a combination of the selective dopamine receptor D2 and D4 inhibitors L-741,626 and L-745,870. This establishes a role for dopamine receptor signaling via G protein βγ subunits in glioblastoma invasion and shows that phosphoinositide 3-kinase mutations in glioblastoma require a context of basal G protein–coupled receptor activity in order to promote this invasion.

## INTRODUCTION

The human brain tumour known as glioblastoma is currently incurable, largely due to its highly invasive nature. Glioblastoma invades surrounding tissue as single cells that often follow preferred anatomical routes including white matter tracts and the outside walls of blood vessels. Multiple studies have suggested a role for the Rac subclass of Rho GTPases in glioblastoma invasion [1–3] (reviewed in [4]). One limitation of these studies is that they were performed in glioblastoma cell lines that are not invasive *in vivo* in animal models. A more recent study has also looked at Rac1 in glioblastoma cells that were isolated under serum-free conditions and therefore retain their *in vivo* invasive properties [5]. This study also identified a role for Rac in invasion using *in vitro* assays. In its active form Rac is bound to GTP. The association with GTP is enhanced by the action of Rac-specific guanine-nucleotide exchange factors (GEFs). Previously the Rac GEFs Trio, Ect2 and Vav3 have been evaluated as candidates for Rac GEFs in glioblastoma [6]. Knockdown of these GEFs reduced the *in vitro* invasive properties of U87MG and SNB19 glioblastoma cell lines. This study did not assess the function of these Rac GEFs in glioblastoma cells that have invasive properties *in vivo* and did not assess a potential role for the Rac GEF PREX1 in glioblastoma invasion.

PREX1 was originally identified in assays testing for Rac activators that were responsive to the PI 3-kinase pathway second messenger phosphatidylinositol 3,4,5 trisphosphate (PIP_3_) [7]. This makes it of particular interest in glioblastoma, as the PI 3-kinase pathway is aberrantly activated in almost all glioblastomas through partial or complete loss of PTEN expression, amplification and/or mutation of growth factor receptors, or mutation of PI 3-kinase itself [8]. The *PREX1* gene encodes a 185 kDa protein that is preferentially expressed in leukocytes and in the brain (reviewed in [9]). A second related gene, PREX2, encodes two proteins, PREX2A and a splice variant, PREX2B. Although initially screened for PIP_3_ responsiveness, PREX1 can be weakly activated either by PIP_3_ binding to its PH domain or by binding of βγ subunits of activated G proteins to its DH domain [7, 10]. Binding of both PIP_3_ and βγ results in full activation of PREX1 in a synergistic fashion [7, 10].

In mice, PREX1 has a physiological role in neutrophils, where it is required for efficient migration and ROS production [11, 12]. PREX1 is also required for efficient neuroblast migration [13]. Given this role in promoting cell motility, PREX1 has been studied for a potential role in cancer cell invasion [14]. Overexpression of PREX1 has been linked to increased migration and metastases in melanoma [15] and prostate cancer [16]. In breast cancer, PREX1 promotes breast cancer metastasis, and also tumour growth, in mouse xenografts [17]. In addition, high expression of PREX1 correlates with decreased disease-free survival in breast cancer patients [18].

We show here that PREX1 is overexpressed in many glioblastomas. As in other cancers, PREX1 promotes motility and invasion in glioblastoma cells. Consistent with current knowledge on the activation of PREX1, glioblastoma invasion requires input from both PI 3-kinase signalling and G protein-coupled receptor signalling. Inhibitor studies identified dopamine receptors as members of the G protein-coupled receptor family that contribute to glioblastoma invasion.

## RESULTS

### PREX1 expression in primary glioblastoma cell cultures

**Figure 1:**
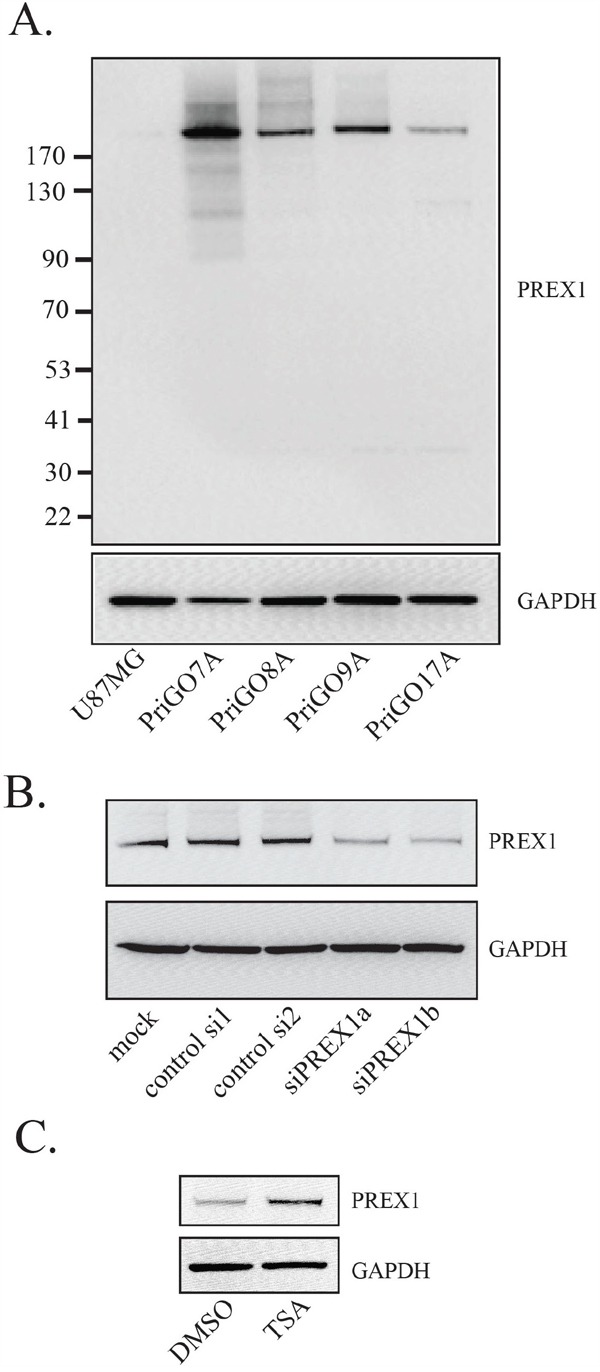
PREX1 expression in glioblastoma cells **A.** PREX1 expression in glioblastoma cell line U87MG and glioblastoma cells isolated from patients under serum-free conditions (PriGO 7A, 8A, 9A and 17A) was analyzed by Western blotting. GAPDH was used as a loading control. **B.** PriGO8A cells were mock transfected or transfected with control siRNA duplex, or with two different siRNA duplexes targeting PREX1 (siPREX1a and siPREX1b). Two days after transfection cell lysates were collected and analyzed by Western blotting. **C.** PriGO17A cells were treated with 100ng/mL histone deacetylase inhibitor Trichostatin A for 24 hours after which cell lysates were collected and analyzed by Western blotting.

PREX1 expression was examined by Western blotting in total cell lysates from glioblastoma cells isolated from patients under serum-free conditions (hereafter referred to as primary glioblastoma cells or PriGO cells). A single band of Mr 184,000 was detected in each primary culture sample, similar to the reported Mr of 186,000 for PREX1 isoform 1 (Figure 1A). Knockdown of PREX1 with two different PREX1-specific duplexes reduced the intensity of this band, indicating that the antibody was specific to PREX1 (Figure 1B). PREX1 expression varied across primary glioblastoma cell cultures from different patients, with the highest expression in PriGO7A cells and the lowest expression in PriGO17A cells (Figure 1A). Expression in primary glioblastoma cell cultures was considerably higher than in the U87MG, DBTRG and A172 human glioblastoma cell lines, where a faint band was detected only with long exposures (Figure 1A and Supplementary Figure 1A). Levels of PREX1 have been reported to be regulated by promoter histone acetylation in prostate cancer [19] and by subtype-specific promoter methylation in breast cancer [20]. To test whether such mechanisms could explain the relatively low expression of PREX1 in PriGO17A cells, these cells were treated with the HDAC inhibitor Trichostatin A. This led to an increase in PREX1 levels in PriGO17A (Figure 1C), supporting a role for histone modifications in controlling PREX1 expression in glioblastoma.

**Figure 2:**
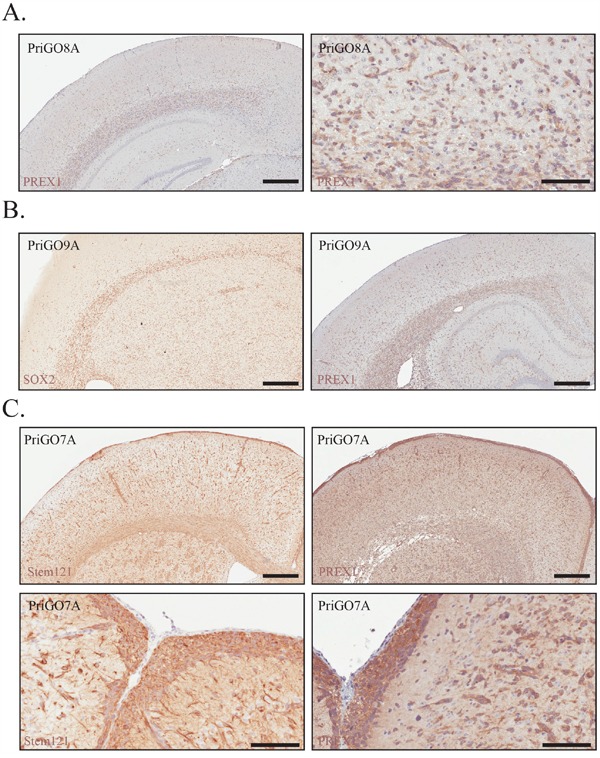
PREX1 expression in glioblastoma tumour xenografts **A.** PriGO8A cells were analysed for PREX1 expression by immunohistochemistry. Left and right panels show low and high magnification images respectively. **B.** PriGO9A cells were injected intracranially in SCID/beige mice and were analysed for PREX1 expression by immunohistochemistry after 6 months of growth (right). SOX2 (left) marks cancer cells. **C.** PriGO7A cells were injected intracranially in SCID/beige mice and were analysed for PREX1 expression by immunohistochemistry after 6 months of growth. Stem121 was used to detect cancer cells. Cancer cell distribution and PREX1 expression is shown at the cortical subarachnoid space (bottom panels). Scale bars are 500μm and 200μm (panel A right image and panel C bottom images).

To investigate the expression of PREX1 in primary glioblastoma cells within their physiological microenvironment, PriGO cells were grown as intracranial xenografts in SCID/Beige mice and analyzed for PREX1 expression by immunohistochemistry. The *in vivo* growth of PriGO8A cells has been described previously [21]. They do not form a distinct tumor mass, but rather grow diffusely in the injected hemisphere with extensive invasion along white matter tracts and invasion of the uninjected hemisphere (reference [21] and Figure 2A). PriGO9A cells show a similar pattern of growth (Figure 2B). PriGO7A cells are also highly invasive, with a greater tendency to invade through the parenchyma and accumulate in the subpial parenchyma (Figure 2C). Intracranial growths of primary glioblastoma cultures showed a uniform expression pattern of PREX1 with the intensity of signal proportional to the Western blot intensities across the PriGO7A, 8A and 9A cell cultures (Figure 2A-2C). Images of the entire brain sections for PREX1 immunohistochemistry of PriGO8A, PriGO9A and PriGO7A xenografts are shown in Supplementary Figure 2.

### PREX1 expression in glioblastoma clinical samples

**Figure 3:**
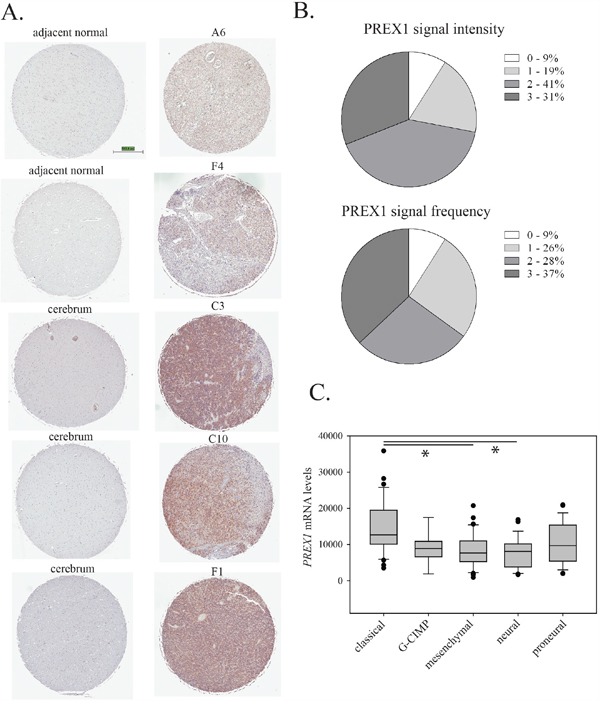
PREX1 expression in glioblastoma clinical cases **A.** PREX1 was analysed by immunohistochemistry of a tissue microarray containing samples from thirty five glioblastoma patients, with adjacent brain tissue from two cases and normal brain tissue from three subjects. Representative stained cores from the two adjacent brain tissue cases and the three normal brain tissue subjects are shown, along with representative cores from five different glioblastoma cases. **B.** Intensity of staining (0 – negative, 1, 2 and 3) and frequency of positive staining (0 – no cancer cells positive, 1 – 0-33%, 2- 33-66% and 3 – 66%-100% cancer cells positive) were scored independently by MD and JW with scoring discrepancies averaged. **C.** PREX1 mRNA expression from the Cell 2013 TCGA RNA Seq V2 dataset was analysed by cancer subtype using cBioPortal [23, 24]. Expression in the classical subtype was significantly different from expression in the mesenchymal and neural subtypes when data were analyzed using Kruskal-Wallis One Way ANOVA on Ranks and All Pairwise Multiple Comparison Procedures (Dunn's Method). * indicates p<0.05.

PREX1 protein expression and distribution within tumour tissue was analysed by immunohistochemistry on a commercial tissue microarray comprised of duplicate cores of 35 individual glioblastoma cases (22 male and 13 female cases), two cases of glioblastoma patient adjacent normal tissue and normal brain samples from three subjects. Each core was scored from 0 to 3 for both the overall intensity of the staining and the frequency of positive signal. Staining of normal tissue and representative examples of staining in tumor tissue are shown in Figure 3A and a graphical summary of the results for tumour tissue is shown in Figure 3B. For normal tissue, two samples of normal brain adjacent to glioblastoma tumours and two samples of normal cerebrum were negative for PREX1. For two additional samples of normal cerebrum, PREX1 was detected, although at low intensity and frequency (Figure 3A). For tumor tissue, ninety-one percent of glioblastoma cases showed some level of PREX1 expression (Figure 3B). As in the PriGO cells, expression varied, with about one third of the cases showing high expression. Analysis of PREX1 mRNA levels in the TCGA database [8, 22] using cBioPortal [23, 24] showed that PREX1 mRNA levels were significantly higher in the classical subtype compared to either the mesenchymal or neural subtype (Figure 3C). Consistent with this, PREX1 mRNA levels are also positively correlated with phosphoEGFR (p=0.021 and p=0.022 for Y1173 and Y1068 phosphorylation, respectively) and EGFR protein expression levels (p=0.028). This analysis also showed that *PREX1* is almost never amplified or mutated in glioblastoma. The association of higher PREX1 levels with the classical subtype fits with the Western blot analysis shown in Figure 1A, as microarray expression analysis of PriGO7A, PriGO8A and PriGO9A cells showed that they are predominantly of the classical molecular subtype, while PriGO17A cells have more mesenchymal subtype characteristics (Kumar *et al*., *submitted for publication*). Data in the TCGA data set were from 96 male and 55 female glioblastoma patients. PREX1 mRNA levels were not significantly different between males and females. PREX1 mRNA levels were significantly higher in patients with MGMT promoter methylation (p = 0.034) but higher levels were not associated with differences in survival.

### PREX1 in glioblastoma invasion and motility

**Figure 4:**
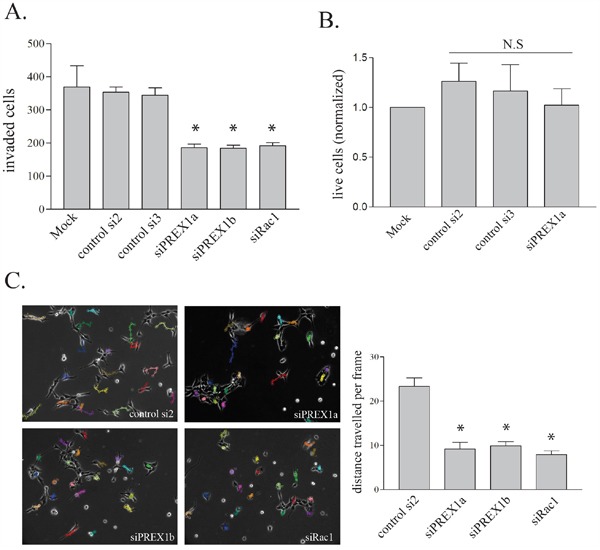
PREX1 in glioblastoma invasion and motility **A.** PREX1 levels were depleted in PriGO 8A cells using siRNA as shown in Figure 1B. In vitro invasion was assessed using Transwell chambers coated with Matrigel basement membrane matrix three days after siRNA-mediated knockdowns. A representative result from three independent experiments for Mock, siControl2, siControl3 and siPREX1a treatments is shown. Data are shown as the mean +/− SE. **B.** Three days after siRNA-mediated knockdowns live PriGO-8A cell counts were quantified by trypan blue exclusion from three independent experiments. Data are shown as the mean +/− SD. **C.** Cell motility was assessed three days after siRNA-mediated knockdowns by video microscopy. Cell movement per ten minute frame was quantified in ImageJ with the MTrackJ plug-in and displayed as the average of twenty cells per condition with each condition done in two to three independent experiments. Data are shown as the mean +/− SE. * P<0.05.

*In vitro* invasion was assessed using Matrigel-coated Transwell chambers. PREX1 knockdown resulted in a significant reduction in the invasiveness of PriGO8A cells (Figure 4A). This was seen with two different RNA duplexes targeting PREX1 and knockdown of PREX1 did not significantly affect cell growth in PriGO8A cells over the time frame of the invasion assays (Figure 4B). A similar reduction in invasion was seen with knockdown of Rac1, indicatting that PREX1 may be a principle activator of Rac1 in these cells (Figure 4A). Loss of invasive behaviour can be a consequence of reduced ability to degrade cell matrix, or a reduction in motility. Motility of PriGO8A cells was determined using time-lapse videomicroscopy and quantified as the average distance travelled by a cell per frame. Knockdown of PREX1 significantly reduced the motility of PriGO8A cells (Figure 4C). As with the invasion assays, knockdown of Rac1 had a similar effect on motility to that seen with knockdown of PREX1 (Figure 4C).

### Upstream regulation of PREX1 activity in glioblastoma

**Figure 5:**
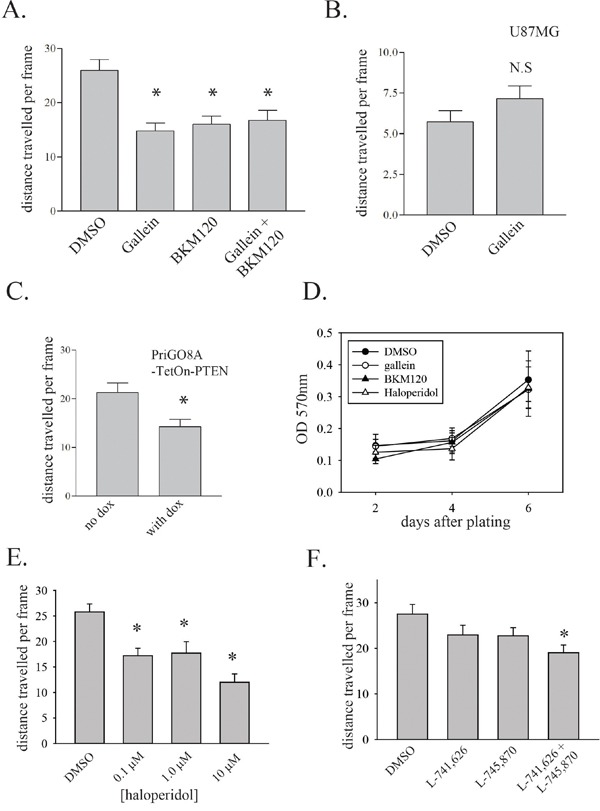
Upstream regulation of PREX1 activity in glioblastoma **A.** PriGO8A cells were treated with 30μM gallein and/or 1μM BKM120 with DMSO used as solvent control. Cell motility was assessed 24 hours after treatment by time-lapse video microscopy as in Figure 4. **B.** U87MG cells were treated with 30μM gallein and 24h later cell motility was assessed by time-lapse video microscopy. **C.** PriGO8A cells transduced with a PTEN cDNA doxycycline inducible lentiviral vector were treated with doxycycline at 1μg/mL for 24 hours in media containing 5% of the standard EGF and FGF2 supplements. Cell motility was assessed by time-lapse video microscopy. **D.** PriGO8A cells were treated with a single dose of gallein, BKM120, haloperidol or DMSO at the concentrations used above. Cell growth was assessed by crystal violet signal intensity (OD 570nm). Data are shown as the mean +/− SD. **E.** PriGO8A cells were treated with the indicated concentrations of haloperidol and 24h later cell motility was assessed by time-lapse video microscopy. **F.** PriGO8A cells were treated with DMSO vehicle, 100 nM L-741,626, 100 nM L-745,870 or a combination of both inhibitors at 100 nM each and 24h later cell motility was assessed by time-lapse video microscopy. For A-C and E-F, data are shown as the mean +/− SE. * P<0.05.

G protein βγ subunits and PIP_3_ binding synergistically activate PREX1 [7]. To inhibit G protein βγ subunit binding and activation of PREX1, the compound gallein was used [25]. Gallein binds purified Gβγ with a Kd of approximately 400 nM at a site that interferes with its interaction with downstream effectors [25]. In addition gallein has been shown to inhibit the Gβγ-dependent activation of Rac1 in neutrophils [25]. Motility of PriGO8A cells was assessed by time-lapse videomicroscopy. Gallein treatment resulted in a significant decrease in migration (Figure 5A). As one control for drug specificity, the same experiment was performed on U87MG cells that have very low level PREX1 expression and presumably rely on other mechanisms for Rac activation. Gallein did not significantly affect the motility of these cells (Figure 5B). BKM120, a selective class I PI3-kinase inhibitor [26], was used to inhibit PIP_3_ generation. Treatment of PriGO8A cells with BKM120 reduced cell motility to the same extent as gallein treatment (Figure 5A). A similar effect on motility was seen when PIP_3_ levels were repressed by expression of PTEN (Figure 5C). A combination of gallein and BKM120 also repressed motility, but this effect was not greater than that seen with either compound alone (Figure 5A). A combination of PREX1 knockdown and gallein treatment did not have a greater effect on motility than PREX1 knockdown alone, consistent with these inhibiting the same pathway (Supplementary Figure 1B). Gallein and BKM120 did not affect the growth of PriGO8A cells at the concentrations used (Figure 5D).

A recent report indicated a role for the dopamine receptor D2 in glioblastoma growth [27, 28]. As this G protein-coupled receptor could be a potential source of G protein βγ subunits for PREX1 activation, the effect of dopamine receptor inhibition with haloperidol was tested. Haloperidol inhibits dopamine receptors D2, D3 and D4 with low nanomolar affinity but at higher concentrations is able to inhibit multiple other G protein-coupled receptors [29, 30]. Testing of the effects of haloperidol at a range of doses showed partial inhibition of motility at 0.1 μM and 1.0 μM (Figure 5E). At a concentration of 10 μM, haloperidol was able to inhibit motility to a similar extent to that seen with knockdown of PREX1. This suggests that both high affinity and low affinity targets contribute to haloperidol-mediated inhibition of motility. To further assess the role of dopamine receptors in glioblastoma cell motility, the effects of the selective dopamine receptor D2 inhibitor L-741,626 and the selective dopamine receptor D4 inhibitor L-745,870 [31, 32] were determined. These experiments were performed using relatively low concentrations (100 nM) of drug to ensure target selectivity. Inhibition of D2 or D4 receptors alone did not significantly reduce motility. However a combination of the two drugs did give significant inhibition, showing that both receptors contribute to glioblastoma motility (Figure 5F).

### PREX1 function in glioblastoma cells from additional patients

**Figure 6:**
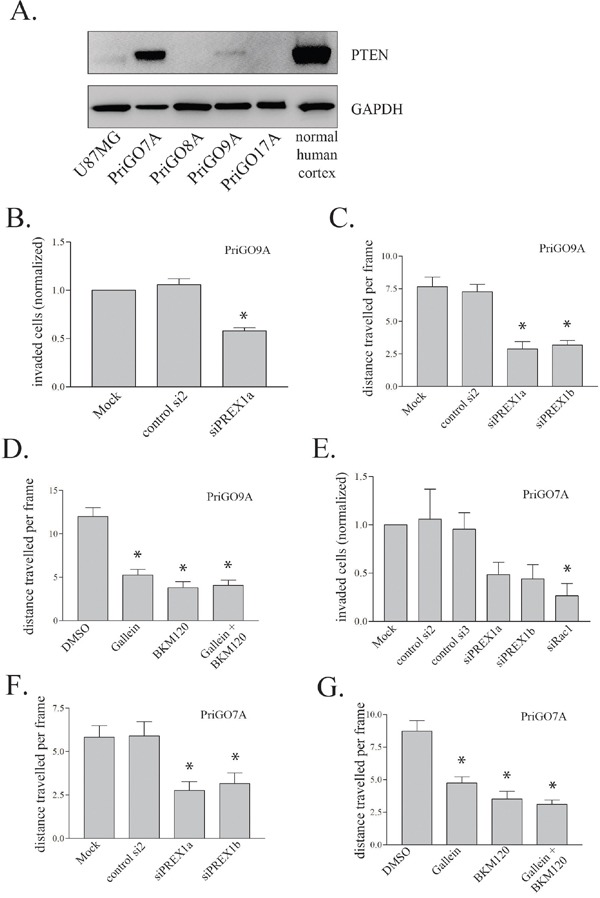
Effect of PREX1 inhibition on PriGO7A and PriGO9A cells **A.** PTEN levels in glioblastoma cells and human cortical tissue lysate were analysed by Western blotting. GAPDH was used as a loading control. **B** and **E**. In vitro invasion as in Figure 4A was performed on PriGO9A and PriGO7A taken as an average of two independent experiments done in duplicate. **C** and **F**. Cell motility of PriGO9A and PriGO7A cell cultures was assessed by time-lapse video microscopy as in Figure 4C three days after PREX1 knockdown by siRNA. **D** and **G**. Cell motility of PriGO9A and PriGO7A cell cultures was assessed by time-lapse video microscopy as in Figure 4C 24 hours after treatment. Data are shown as the mean +/− SE. * P<0.05.

To assess the generalizability of the above findings, PREX1 function was assessed in glioblastoma cells from two additional patients, PriGO9A and PriGO7A. As mentioned above, PriGO8A, PriGO9a and PriGO7A cells all cluster with the “classical” molecular subtype (Kumar *et al., submitted for publication*), which was the subtype identified here as having generally higher levels of PREX1. These cells differ in their PTEN status, which could potentially influence their ability to activate PREX1. PriGO8A cells lack any detectable expression of PTEN [33]. PriGO9A cells show a faint PTEN band on Western blots (Figure 6A). Immunofluorescence showed that this is due to a small subset of cells that express PTEN (not shown). Thus PriGO9A cells are a mixed culture of PTEN-null cells and cells that are probably only haploinsufficient for PTEN. Knockdown of PREX1 decreased both invasion and motility in PriGO9A cells, similar to what was seen in PriGO8A cells (Figure 6B and 6C). Blockade of PREX1 input signals with gallein and BKM120 also had similar effects in PriGO9A cells to those seen in PriGO8A cells (Figure 6D). PriGO7A cells show a strong PTEN band (Figure 6A). Comparison with normal human brain tissue shows that this band is roughly half the intensity of that seen in normal brain. Thus PriGO9A cells are likely haploinsufficient for PTEN. Knockdown of PREX1 in these cells also inhibited invasion and motility, as did treatment with gallein and BKM120 (Figure 6E-6G). PREX1 therefore has a role in glioblastoma motility in cells from multiple patients, at least within the classical molecular subtype, and this is independent of whether cells have partially or fully compromised PTEN function. Inhibition of dopamine receptors D2 and D4 also caused a significant decrease in motility of PriGO9A and PriGO7A cells, similar to what was seen in PriGO8A cells (Supplementary Figure 1C and 1D).

### Signalling downstream of PREX1

**Figure 7:**
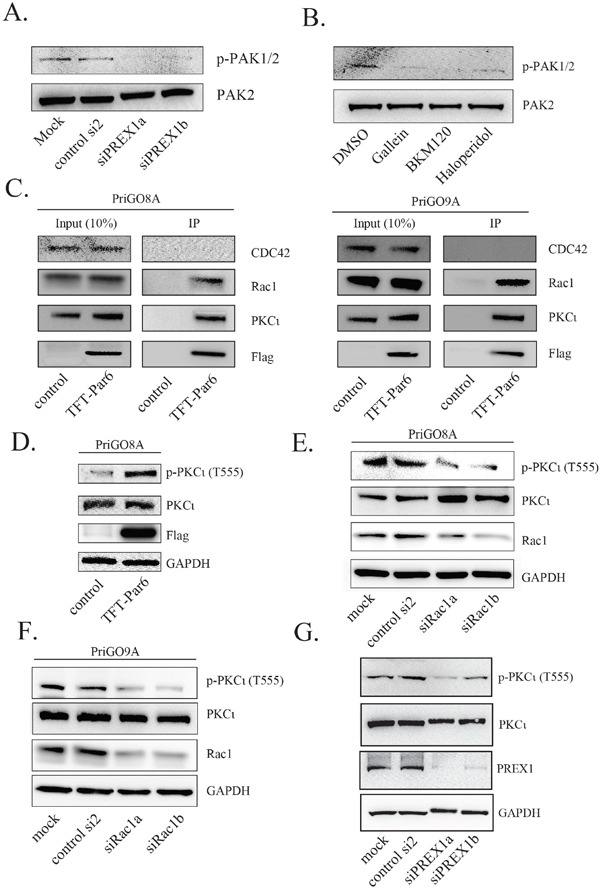
PREX1 signals through RAC1 to regulate PAK and PKCι activity **A.** Two days after PREX1 knockdown using siRNA phospho-PAK1/PAK2 levels and total PAK2 levels were analysed by Western blotting. **B.** PriGO8A cells were treated with DMSO, gallein, BMK120 and haloperidol as previously for 24 hours. Lysates were collected and analyzed for phospho-PAK1/PAK2 and total PAK2 levels by Western blotting. **C.** PriGO8A (left) and PriGO9A cells (right) were transduced with triple-flag-tagged Par6A (TFT-Par6). Lysates were collected under non-denaturing conditions and immunoprecipitation was performed using anti-flag M2-conjugated magnetic beads. Bound proteins were eluted using triple-flag-peptide. **D-G.** Phospho-PKCι (T555) levels were analysed by Western blot following expression of TFT-Par6 (D), knockdown of Rac1 (E and F) and knockdown of PREX1 (G).

Rac1 activation leads to the downstream activation of multiple kinase signalling pathways, either directly or indirectly [34]. GTP-Rac directly binds to and activates PAK1, PAK2 and PAK3 kinases, which control multiple aspects of cell behaviour including F-actin polymerization [35]. The activation of PAK1/2 by Rac can be monitored by the autophosphorylation of PAK1/2 on S144/S141 [36]. Knockdown of PREX1, as well as treatment of cells with gallein, BKM120 and haloperidol, all decreased PAK1/2 autophosphorylation (Figure 7A and 7B), showing that PREX1 has a significant role in the activation of this pathway.

GTP-bound Cdc42 and Rac1 also have the potential to interact with the polarity protein Par6 to influence the activity of atypical PKC in polarity complexes [37, 38]. Of the three Par6 isoforms, Par6A has the highest expression in normal brain and in glioblastoma, based on data in the Human Protein Atlas (www.proteinatlas.org) [39]. A flag-tagged Par6A lentiviral expression vector was constructed and transduced into PriGO8A and PriGO9A cells. Pull down experiments showed that Par6 associates with PKCɩ as expected and preferentially associates with Rac1 rather than Cdc42, even though both are expressed in these cells (Figure 7C). Par6A itself functions as an activator of atypical PKC in these cells, as its overexpression increased phosphorylation of the atypical protein kinase C PKCɩ at Thr555 (Figure 7D). Phosphorylation at this site in the turn motif of PKCɩ stabilizes it in an active conformation [40]. Knockdown of Rac1 in both PriGO8A and PriGO9A cells led to a reduction in PKCɩ Thr555 phosphorylation (Figure 7E-7F). Knockdown of PREX1 also decreased PKCɩ Thr555 phosphorylation (Figure 7G). PREX1 is therefore able to influence PKCɩ activity, likely through the interaction of Rac1 with Par6.

## DISCUSSION

This study shows that PREX1 is overexpressed in many glioblastomas. Bioinformatics analysis showed that high level expression of PREX1 mRNA is associated with the classical molecular subtype. PREX1 expression is preserved in glioblastoma cells isolated from patients under serum-free conditions and was higher in glioblastoma cells of the classical subtype (PriGO8A, PriGO9A and PriGO7A) than those showing a mesenchymal subtype component (PriGO17A cells). PREX1 was required for glioblastoma cell invasion in the three glioblastoma cell cultures with a predominantly classical subtype expression signature. PREX1 therefore has a general role in glioblastoma invasion, although it is possible that some glioblastomas use an alternate mechanism. PREX2 appears to be expressed at much lower levels in glioblastoma based on microarray expression analysis (Kumar *et al*., *submitted*) and therefore seems less likely to have a role in invasion.

There are several important consequences regarding the use of PREX1 as a GEF for Rac in glioblastoma. First, this provides a direct link between Rac and activating mutations in the PI 3-kinase pathway, which are very common in glioblastoma. In particular, *PTEN* loss is very common, with loss of at least one copy of PTEN occurring in over 80% of glioblastomas. No mutations have been reported in G protein signalling pathways in glioblastoma, although these have been reported in other cancer types [41]. This is consistent with there already being constitutive G protein signalling in these cells. G protein signalling can lead to PIP3 generation through the activation of PI 3-kinase p110β and p110γ. The latter is absent in the primary glioblastoma cell lines used here, but p110β is relatively abundant. *PTEN* at normal copy number may be sufficient to suppress PIP3 generated through p110β. Constitutive G protein signalling may provide an explanation as to why *PIK3CA* mutations are relatively common in glioblastoma (9% in the TCGA database) while *PIK3CB* mutations are rare (1% in the TCGA database). It would be expected that *PIK3CB* mutations would be rare if there is already constitutive signalling through Gβγ-activated p110β, as this would result in a lack of selection pressure for *PIK3CB* mutations.

A second aspect of PREX1 use is that this may provide a signalling mechanism that is highly sensitive to small changes in PIP3 levels. Binding of Gβγ and PIP3 to PREX1 activate it synergistically. In the context of constitutive Gβγ signalling this creates a system that will be hyper-responsive to changes in PIP3 levels. In gliomagenesis, loss of one copy of *PTEN* is thought to be an early driver event [42]. While this might be expected to cause only a small increase in PIP3 levels, the hypersensitivity of PREX1 responses in a background of constitutive G protein signalling could convert this into substantial downstream effects. These potentially include effects on cell motility through the p21-activated serine-threonine kinases and PKCɩ, as well as effects on differentiation status mediated by PKCɩ [21, 33]. While the focus of this study is on glioblastoma, other studies have pointed to an apparent high sensitivity to small changes in PIP3 levels in other systems [43]. This may also be explained by PIP3 activation of PREX1 that is “primed” by Gβγ activity arising from constitutive G protein-coupled receptor signaling.

Li *et al*. have described a mechanism in which cooperative signalling between EGFR and the dopamine receptor D2 drive glioblastoma growth [27]. This effect was due in part to EGFR and dopamine receptor D2 signalling converging on the Ras/ERK pathway. PREX1 provides a second mechanism by which these signals can be integrated, in this case to drive invasion. Haloperidol was used in this study as an initial step to identify G protein-coupled receptors that are involved in PREX1 activation. Haloperidol is a dopamine receptor D2 inhibitor, although it also inhibits dopamine receptors D3 and D4 [30], the alpha-1A adrenergic receptor and, at higher doses, the 5HT_2A_ receptor [29]. Microarray expression analysis of the PriGO cells used here (Kumar *et al*., *submitted for publication*) shows that they express negligible levels of the latter two receptors, suggesting that dopamine receptors are the relevant target. A specific role for dopamine receptors D2 and D4 was supported by the use of highly selective inhibitors, which caused a significant inhibition of motility when used in combination. However this inhibition was only partial and it is likely that glioblastoma cells are able to use multiple G protein-coupled receptors to activate PREX1. Activation of G protein-coupled receptors in glioblastoma cells could occur if the cells produce their own agonists such as dopamine. However this is not essential, as there is evidence for basal activity of G protein receptors, including dopamine receptors, in the absence of agonist [44, 45]. G protein-coupled receptors appear to spontaneously switch between inactive and active conformations, with agonists stabilizing the latter and inverse agonists stabilizing the former. Haloperidol and L-741,626 both function as inverse agonists, repressing both agonist-activated and basal dopamine receptor signaling [46, 47].

The findings here, along with the findings of Li *et al*. [27] and a more recent study on dopamine receptor D4 function in glioblastoma [48], suggest that further studies characterizing the G protein-coupled receptors involved in promoting glioblastoma growth and invasion may lead to novel strategies for treating this disease.

## MATERIALS AND METHODS

### Antibodies and reagents

PREX1 [D8O8D] rabbit monoclonal, PTEN [138G6] rabbit monoclonal, Phospho-PAK1 (Ser144)/PAK2 (Ser141) rabbit polyclonal, PAK2 [3B5] mouse monoclonal were purchased from Cell Signaling (Danvers, MA, USA). GAPDH [6C5] mouse monoclonal and CDC42 [M152] mouse monoclonal were purchased from Abcam (Cambridge, MA, USA). Phospho-PKCι (T555)/PKCλ (T563) rabbit polyclonal was purchased from Invitrogen (Carlsbad, CA, USA) and PKCι mouse monoclonal from (BD Transduction Laboratories (Mississauga, ON, Canada). Sox2 mouse monoclonal was from R&D Systems (Minneapolis MN, USA). Rac1[23A8] mouse monoclonal, anti-Flag M2 mouse monoclonal and Stem121 mouse monoclonal were purchased from Millipore (Temecula, CA, USA), Sigma-Aldrich (Oakville, ON, Canada) and StemCells Inc. (Newark, CA, USA), respectively. Human brain cerebral cortex protein medley was purchased from Clontech (Mountain View, CA, USA). The following inhibitors were used in the study: gallein (Santa Cruz Biotechnology, CA, USA), BKM120 (Sigma-Aldrich, Oakville, ON, Canada); trichostatin A (Cayman Chemical Company, Ann Arbor, MI, USA); haloperidol, L-741,626 and L-745,870 (Tocris Bioscience, Minneapolis, MN, USA).

### Cell culture

Primary glioblastoma (PriGO) cultures were isolated following a protocol approved by the Ottawa Health Science Network Research Ethics Board as described previously [33] and were grown on laminin-coated plates in Neurobasal A medium supplemented with B27, N2, EGF and FGF2 at 37°C in 5% O_2_. PriGO cells were genetically modified with lentiviral vectors as described previously [33]. U87MG, DBTRG and A172 cells were all from the ATCC (Manassas VA, USA).

### Mouse intracranial xenografts

Animal procedures were approved by the Animal Care Committee at the University of Ottawa. PriGO cell culture cells were established as xenografts in SCID/Beige mice (Charles River Laboratories, MA, USA) as described previously [21]. 1×10^6^ cells in 10μL Neurobasal A medium were injected intracranially using a Hamilton 700 series syringe (Reno, NV, USA) in a stereotactic system. Mice were monitored for signs of morbidity or six months of intracranial growth prior to endpoint. Whole brains were harvested, formalin fixed and paraffin embedded. Immunohistochemistry for the human-specific antigen STEM121was performed as described previously [21]. For PriGO9A cells, immunohistochemistry for Sox2 was performed instead of STEM121, as these cells do not express the STEM121 antigen.

### RNA interference

RNA duplexes were from Dharmacon (Lafayette, CO, USA) and had the following sense strand sequences: siPREX1a (GAGAUGAGCUGCCCUGUGA), siPREX1b (GAAAGAAGAGUGUCAAAUC), siRac1a (UAAGGAGA UUGGUGCUGUA), siRac1b (UAAAGACACGAUC GAGAAA), siDRD2a CCUGAGGGCUCCACUAAAG, siDRD2b GUAGGUGAGUGGAAAUUCA. Non-Targeting siRNA #2 and #3, also from Dharmacon, were used as controls. RNA duplexes were incubated with PriGO cells at a concentration of 40nM in a mixture of Oligofectamine, Optimem and Neurobasal A medium. After 48 hour incubation, Neurobasal A media was replenished and cells were used for subsequent experiments.

### *In vitro* invasion

After RNAi-mediated gene knockdown, PriGO cell cultures were counted and re-plated in the top compartment of 8μm Transwell inserts with Matrigel (Corning BioCoat, Corning, NY, USA) and in parallel in a 24 well plate with laminin directly added to the top and bottom compartments. 20-24 hours later, cells remaining in the top compartment were scraped off with a swab and invaded cells were fixed and stained using the Kwik Diff staining kit (ThermoElectron, Pittsburgh, PA, USA). Migrated cells were counted within 5 random fields at 40x magnification.

### Cell counts

Cell counts following siRNA knockdowns were analyzed using the Invitrogen Countess II instrument (Thermo Fisher Scientific, Waltham, MA, USA). Cell counts following inhibitor treatment were determined by crystal violet stain intensity at 570nm using the Multiskan Ascent plate reader with the Ascent Software program (Thermo Fisher Scientific, Waltham MA, USA).

### Videomicroscopy

Cells were directly plated on laminin coated Bioptechs delta-T dishes (Butler, PA, USA) in 1mL media. During image acquisition cells were maintained in sealed chambers at 37°C. Phase-contrast images were taken at 10 minute intervals for 1-1.3 hours using a 10x objective. Images were acquired using a ZiessAxiovert 200 M microscope equipped with a AxioCamHRm CCD camera (Zeiss, Göttingen, Germany). Motility was quantified using the MtrackJ plugin [49] in ImageJ software (National Institutes of Health, Bethesda, Maryland, USA) and scored as the average distance to point per cell per frame.

### Tissue microarray analysis

A glioblastoma tissue microarray consisting of 35 cores of glioblastoma tissue, two cores of adjacent brain tissue and three cores of normal brain tissue, all in duplicate, was purchased from US Biomax (GL805a F113, Rockville, MD, USA). Immunohistochemistry was done as described previously [21]. Antigen retrieval was performed in citrate buffer, pH 6.0 (Vector, Burlingame, CA, USA) in a decloaking chamber using the instrument's default program (Biocare Medical, CA, USA). Slides were incubated with PREX1 [D8O8D] rabbit monoclonal at a concentration of 4 ug/ml at 4°C overnight. The DakoEnVision+ system HRP labeled polymer was used for detection of bound antibody (Dako North America, Carpinteria, CA, USA) and sections were developed using DAB Peroxidase Substrate Kit (Vector, Burlingame, CA, USA) and counterstained with haematoxylin (Vector; Burlingame, CA, USA). Slides were digitized using the ScanScope CS2 (Aperio, CA, USA). The tissue microarray was scored independently by MD and JW and discrepancies were averaged. Signal intensity per core was scored as 0, 1, 2, or 3. Frequency of positive staining per core was scored as 0 (0% of cancer cells positive), 1 (0% to 33% of cancer cells positive), 2 (33% to 66% of cancer cells positive), and 3 (66% to 100% of cancer cells positive).

### Immunoprecipitation

PriGO cells transduced with triple-flag-tagged Par6A were scraped and collected in ice cold PBS buffer. Cells were then pelleted and resuspended in 1 ml of lysis buffer (20 mM Tris-HCl, 150 mM NaCl, 1% Triton X-100, 20 mM NaF, 1 mM Na_3_VO_4_, 1mM glycerol-2-phosphate, 1 mM benzamidine, 1 mM β-mercaptoethanol, and 1 μg/ml each of leupeptin, pepstatin, and aprotinin at pH 7.5). Cells were passed once through a 27-gauge needle and centrifuged. Total protein was measured by BCA assay and equal amounts were used in immunoprecipitations. Cell lysates were incubated with anti-flag M2 magnetic beads (Sigma-Aldrich, Oakville, ON, Canada) for three hours with gentle rocking at 4°C. Beads were trapped with a magnet and washed three times with lysis buffer. For elution, lysates were incubated with 200μg/mL triple flag peptide (Sigma-Aldrich, Oakville, ON, Canada) for three hours.

### Statistical analyses

All statistical analyses were performed using SigmaPlot12 software. Data was tested for normal distribution using the Shapiro-Wilk test. For data that was not normally distributed, statistical significance was determined using either the Mann-Whitney Rank Sum test for comparison of two groups or Kruskal-Wallis One Way Analysis of Variance on Ranks and All Pairwise Multiple Comparison Procedures (Tukey Test) for comparison of multiple groups. For normally-distributed data, statistical significance was determined using two-tailed t-tests for comparison of two groups or One Way Analysis of Variance for comparison of multiple groups. A p value less than 0.05 was considered significant.

## SUPPLEMENTARY MATERIALS FIGURES AND TABLES



## Abbreviations

GEF: guanine nucleotide exchange factor
