# Supplementary material for: PREX1 integrates G protein-coupled receptor and phosphoinositide 3-kinase signaling to promote glioblastoma invasion
Source: Oncotarget. 2016 Dec 29;8(5):8559–73. doi: 10.18632/oncotarget.14348 (PMC5352422; doi:10.18632/oncotarget.14348)
Supplement: Supplementary file 1 [file oncotarget-08-8559-s001.pdf]

PREX1 integrates G protein-coupled receptor and phosphoinositide 3-kinase signaling to promote glioblastoma invasion

SUPPLEMENTARY MATERIALS

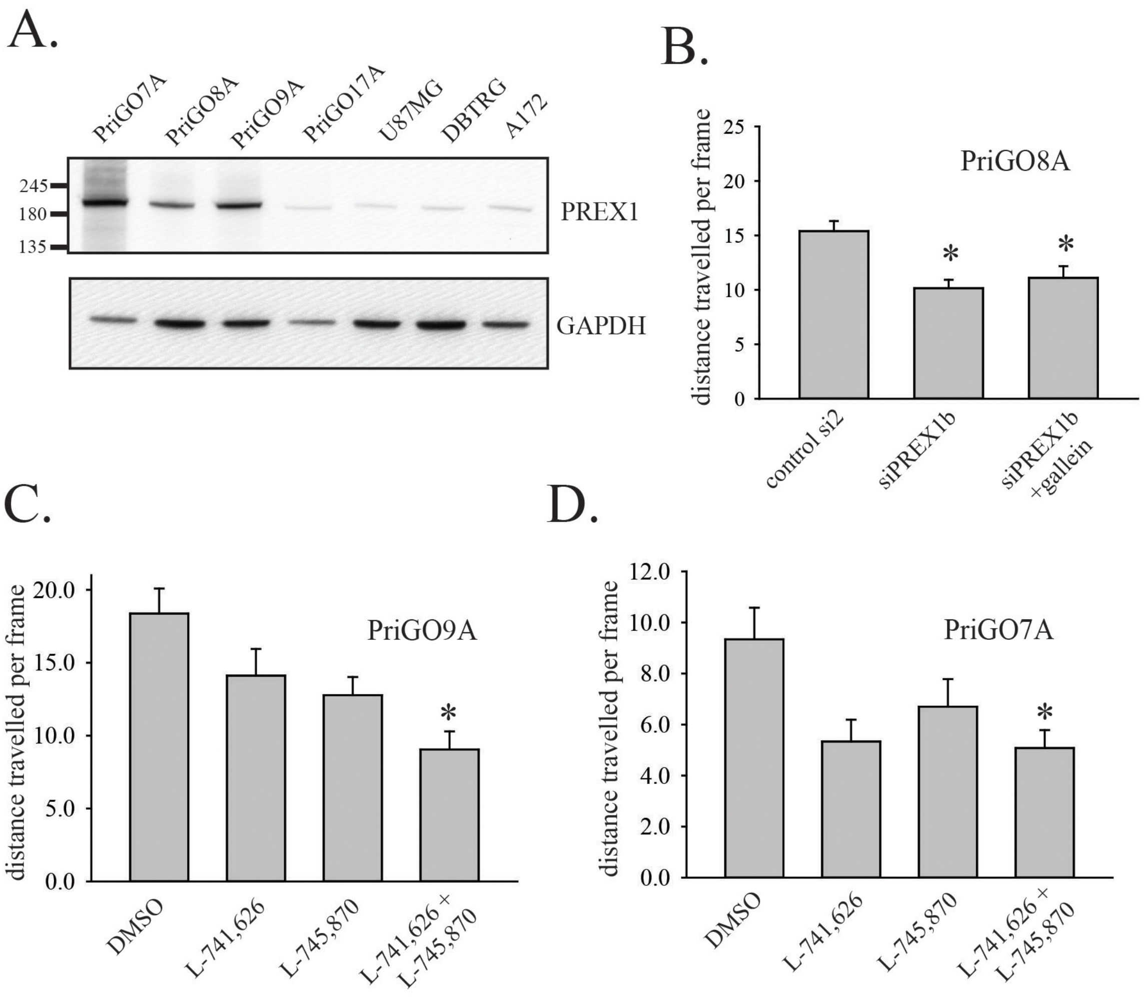

Supplementary Figure 1: A. PREX1 expression in additional glioblastoma cell lines. PREX1 expression in glioblastoma cell line U87MG, DBTRG and A172 and glioblastoma cells isolated from patients under serum-free conditions was analyzed by Western blotting. Weak expression of PREX1 is detected in the cell lines with a longer exposure than shown in Figure 1. GAPDH was used as a loading control. **B.** Effects of combined PREX1 knockdown and gallein of PriGO8A motility. Cell motility was assessed three days after siRNA-mediated knockdown. Gallein was added 24 h before assessment of cell motility. The siPREX1b and siPREX1b + gallein conditions were not significantly different from each other. **C** and **D.** Effects of dopamine receptor inhibition on motility of PriGO9A (C) and PriGO7A (D) cells. PriGO9 and PriGO7A cells were treated with DMSO vehicle, 100 nM L-741,626, 100 nM L-745,870 or a combination of both inhibitors at 100 nM each and 24h later cell motility was assessed by time-lapse video microscopy. Data for B-D are shown as the mean +/- SE. * P<0.05 relative to control si2 or DMSO.

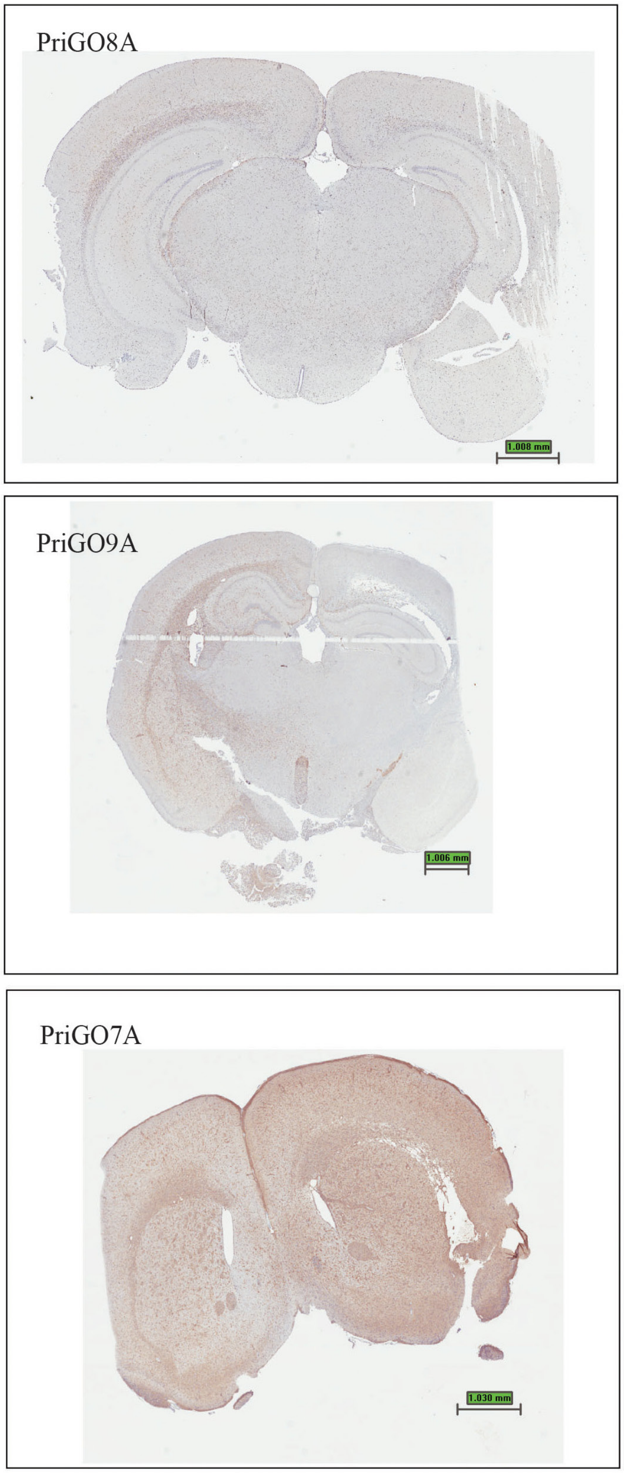

Supplementary Figure 2: PREX1 expression in glioblastoma tumour xenografts: Images of whole brain sections. PriGO8A (top), PriGO9A (middle) and PriGO7A (bottom) cells were analysed for PREX1 expression by immunohistochemistry as described in Figure 2. The scale bar is 1 mm.
